# Comparison of Potentially Preventable Hospitalizations Related to Diabetes Among Native Hawaiian, Chinese, Filipino, and Japanese Elderly Compared with Whites, Hawai‘i, December 2006–December 2010

**DOI:** 10.5888/pcd10.120340

**Published:** 2013-07-25

**Authors:** Tetine L. Sentell, Hyeong Jun Ahn, Deborah T. Juarez, Chien-Wen Tseng, John J. Chen, Florentina R. Salvail, Jill Miyamura, Marjorie L.M. Mau

**Affiliations:** Author Affiliations: Hyeong Jun Ahn, Chien-Wen Tseng, John J. Chen, Marjorie L.M. Mau, John A. Burns School of Medicine, Honolulu, Hawai‘i; Deborah T. Juarez, University of Hawai‘i at Hilo, Honolulu, Hawai‘i; Florentina R. Salvail, Hawai‘i Department of Health, Honolulu, Hawai‘i; Jill Miyamura, Hawai‘i Health Information Corporation, Honolulu, Hawai‘i.

## Abstract

**Introduction:**

Approximately 25% of individuals aged 65 years or older in the United States have diabetes mellitus. Diabetes rates in this age group are higher for Asian American and Pacific Islanders (AA/PI) than for whites. We examined racial/ethnic differences in diabetes-related potentially preventable hospitalizations (DRPH) among people aged 65 years or older for Japanese, Chinese, Filipinos, Native Hawaiians, and whites.

**Methods:**

Discharge data for hospitalizations in Hawai‘i for people aged 65 years or older from December 2006 through December 2010 were compared. Annual rates of DRPH by patient were calculated for each racial/ethnic group by sex. Rate ratios (RRs) were calculated relative to whites. Multivariable models controlling for insurer, comorbidity, diabetes prevalence, age, and residence location provided final adjusted rates and RRs.

**Results:**

A total of 1,815 DRPH were seen from 1,515 unique individuals. Unadjusted RRs for DRPH by patient were less than1 in all AA/PI study groups compared with whites, but were highest among Native Hawaiians and Filipinos. In fully adjusted models accounting for higher diabetes prevalence in AA/PI groups, Native Hawaiian (adjusted rate ratio [aRR] = 1.59), Filipino (aRR = 2.26), and Japanese (aRR = 1.86) men retained significantly higher rates of diabetes-related potentially preventable hospitalizations than whites, as did Filipino women (aRR = 1.61).

**Conclusion:**

Native Hawaiian, Filipino, and Japanese men and Filipino women aged 65 years or older have a higher risk than whites for DRPH. Health care providers and public health programs for elderly patients should consider effective programs to reduce potentially preventable hospitalizations among Native Hawaiian, Filipino, and Japanese men and Filipino women aged 65 years or older.

## Introduction

Approximately 25% of the elderly (aged ≥65 years) in the United States have diabetes mellitus (1). Diabetes rates in this age group are higher for Asian Americans and Pacific Islanders (AA/PI) than for whites (2). For instance, during 2005 through 2007, Native Hawaiian elderly had twice the rate of diabetes (25.4%) of that for whites (13.1%) (3). Diabetes incidence among older Asians is increasing faster than in many other racial groups (4). In one study, the adjusted diabetes prevalence rates for Asians aged 67 years or older increased 68.0%, from 144 per 1,000 to 243 per 1,000, over a 7-year period; this was the highest increase among all racial/ethnic groups studied (4). Disparities in diabetes-related complications are also seen for Pacific Islanders compared with whites (2,5). Asians also show higher rates of some types of diabetes-related complications, including end-stage renal disease (6).

Little is known about diabetes-related potentially preventable hospitalizations (DRPH) in AA/PI groups. Because diabetes can typically be managed in ambulatory care settings, most hospitalizations for diabetes-related conditions are considered potentially preventable (7). Decreasing such preventable hospitalizations can affect 4 key health care goals — reducing cost, improving quality, increasing health equity, and relieving suffering — and are a focus of many recent policy initiatives (7,8).

Many AA/PI groups have limited access to high-quality, culturally relevant primary care and diabetes care (2); thus, AA/PI elderly may not only have higher diabetes prevalence than whites but also higher rates of DRPH. Preventable hospitalizations are known to be higher among black and Hispanic elderly than for whites (9), but detail is lacking for AA/PI elderly, particularly among specific AA/PI subpopulations. This is mainly due to the small sample sizes of AA/PI in many studies, which preclude meaningful AA/PI disaggregation. The few studies on preventable hospitalizations that do include AA/PI groups typically combine these diverse and heterogeneous subgroups into 1 category (10,11) despite known limitations of this approach (2,12,13).

Studies aggregating AA/PI populations have found lower rates of potentially preventable hospitalizations than among whites in elderly and nonelderly populations (10,11). However, evidence suggests that AA/PI subgroup variations in DRPH are hidden by this categorization method (2,12,13). In particular, DPRH may be higher in Native Hawaiians and Filipinos, groups with known health disparities and poorer access to care compared with white and other AA/PI groups (2,12). This hypothesis is supported by a recent study considering all preventable hospitalizations across disaggregated AA/PI subgroups that found strong evidence of disparities for some AA/PI subgroups (14). However, this study did not focus in particular on DRPH or the elderly and did not control for other factors that are known to vary by AA/PI subgroups (2,12) and might impact DRPH (such as sex or insurance status).

The goal of this study was to investigate disparities in DRPH for elderly AA/PI groups compared with whites by using data from all hospitalizations in Hawai‘i over 4 years, from December 2006 through December 2010.

## Methods

Hawai‘i is home to 29% of the total US Native Hawaiian or other Pacific Islander population (15), and more than 40% of the population in Hawai‘i identifies as Asian (16). As such, hospital data in Hawai‘i has unique racial and ethnic detail about AA/PI groups not available in other states. We analyzed Hawai‘i Health Information Corporation (HHIC) inpatient data for December 2006 through December 2010; HHIC data have detailed discharge data from all hospitalizations by all payers (17), including patient race/ethnicity, insurer, age, sex, and *International Classification of Diseases, Ninth Revision, Clinical Modification* (ICD-9) primary diagnosis, secondary diagnosis, and procedure codes.

### Sample

All hospitalizations of those aged 65 years or older were considered (N = 167,793). Hospitalizations with Department of Defense (DOD) as payer were excluded (n = 7,987) because the DOD does not consistently report race/ethnicity data. Hospitalizations without valid race/ethnicity data were excluded (n = 3,073) as were those not reporting a race/ethnicity for the 5 largest ethnic subgroups in Hawai‘i (ie, Japanese, Chinese, Native Hawaiians, Filipinos, or white), which were the focus of this study. To be consistent with population and disease prevalence totals used for rate denominators (estimated for Hawai‘i residents), individuals not from Hawai‘i were excluded (n = 5,362). We also excluded transfers and unknown admission source (n = 4,517) to meet the definitions of the Agency for Healthcare Research and Quality (AHRQ) for diabetes-related preventable hospitalizations.

HHIC data include a master patient identification variable that tracks individuals across all hospitals in the state. Considering unique individuals confirms that multiple visits by members of certain racial/ethnic groups are not driving health disparities, an important issue in diabetes where racial disparities are seen in readmissions (18). After exclusions, there were a total of 146,854 eligible hospitalizations from 72,876 unique patients.

### Diabetes-related potentially preventable hospitalizations

We followed AHRQ definitions to identify DRPH (19). Specifically, we included 1) uncontrolled diabetes without mention of a short-term or long-term complication, 2) diabetes with short-term complications (eg, ketoacidosis, hyperosmolarity, coma), 3) diabetes with long-term complications (eg, renal, eye, neurologic, circulatory, or complications not otherwise specified), and 4) lower extremity diabetes-related amputations. More detail is available from AHRQ (www.qualityindicators.ahrq.gov/Downloads/Modules/PQI/V31/pqi_guide_v31.pdf).

### Race/ethnicity

The HHIC race/ethnicity variable was created from race/ethnicity categories available consistently across all hospitals in Hawai‘i from December 2006 through December 2010 (17). Race/ethnicity data are typically provided by patient self-report at intake and include only 1 primary race. Mixed-race individuals are represented as their primary race of identification or excluded by being part of the “other” racial/ethnic category.

### Control variables

In multivariable models, we included sex, because diabetes prevalence varies by this factor (20); comorbidity, defined by the Charlson comorbidity index (CCI) (21); payer (Medicare, Medicaid, private, and other); location of residence (lives on Oahu vs another Hawaiian island), based on evidence that access to care is often worse on the other islands compared with the more urban Oahu; and age (continuous), because of differences in age structure by racial/ethnic groups. In summary tables, unadjusted and adjusted rates are presented by sex to be more useful for clinical and policy purposes.

### Denominators

When calculating rates of preventable hospitalizations, population totals within the relevant geographic region (eg, the state) are often used as denominators (22). This takes into account the fact that subpopulations have different sizes, allowing identification of the burden of preventable hospitalizations in specific subpopulations. Recent research has emphasized that the disease burden by subpopulations should also be considered in preventable hospitalizations rates (22), particularly when there are known disparities in disease prevalence across subgroups (23). To understand the full burden of DRPH specifically among each racial/ethnic subgroup in Hawai‘i, we calculated DRPH rates first using population totals, and then using disease prevalence totals.

Population totals by sex and race/ethnicity combinations as well as the number of people with diabetes by subgroup were obtained from 2007–2010 Hawai‘i Behavioral Risk Factor Surveillance System (BRFSS) data. The BRFSS is the standard for state-level diabetes rates in Hawai‘i. Self-reported diabetes prevalence was calculated yearly from this representative statewide survey using standard methods (24). Four years of survey data were combined to provide more reliable state-level estimates of disease burden by race/ethnicity. The BRFSS race/ethnicity classification scheme is compatible with that used by HHIC because it is self-reported and includes racial/ethnic categories for the 5 major racial/ethnic groups in Hawai‘i.

### Statistical analysis

Characteristics of the patients with a DRPH were summarized by descriptive statistics for each racial/ethnic subgroup and compared among subgroups using χ^2^ tests or Fisher’s exact tests (for categorical variables) and analysis of variance (ANOVA) or nonparametric Kruskal–Wallis test (for continuous variables). For patients with multiple visits, we used the patient’s first hospitalization in the analysis.

The unadjusted average annual rates of DRPH by patient among AA/PI subgroups and whites were calculated by sex first by using BRFSS population totals and then using population-level totals of diabetes prevalence as denominators. Unadjusted rate ratios (RR) of DRPH by patient were then calculated by dividing the unadjusted rate for each racial/ethnic group by the unadjusted rate for whites. A possible disparity for an AA/PI subgroup relative to whites is represented as RR greater than 1.0. Next, multivariable models were developed to estimate diabetes-related potentially preventable hospitalization rates by patient adjusting not only for sex and race/ethnicity but also for other explanatory factors that may predict hospitalization (ie, comorbidity, residence in Oahu, age, and insurer). To fully understand the portrait of DRPH by racial group, we used 2 multivariable models. In the first model (model A), adjusted rates were calculated by racial/ethnic group by using population totals for rate denominators. This represents the most common portrait of potentially preventable hospitalizations and matches AHRQ guidelines. In the second model (model B), adjusted rates were calculated by racial/ethnic group by using diabetes prevalence for the rate denominators. This represents a portrait of preventable hospitalizations that may more fully capture disparities and shed more light on access to care or quality of outpatient care issues.

In both models, percentage of public insurance (Medicare and Medicaid) and median CCI for each race and sex combination were calculated considering insurer and comorbidity factors, respectively, and entered into models. Overall hospitalization data were modeled using negative binomial regressions models adjusting for possible overdispersion (25). Multivariable adjusted rate ratios (aRR) of patients with DRPH by patient were derived from the final models for each racial/ethnic group compared with whites. All data analyses were performed in SAS 9.3 (SAS Institute Inc, Cary, North Carolina). A 2-tailed *P* value of less than .05 was regarded as significant.

## Results

A total of 1,815 DRPH by 1,515 unique individuals were found (Table 1). Japanese represented the highest percentage (34.8%) of total DRPH followed by Filipinos (20.3%), whites (20.1%), Native Hawaiians (18.2%) and Chinese (6.6%). Across racial groups, no significant differences were seen in the average number of DRPH per person or the percentage of DRPH by payer. Most DRPH (>90%) were paid by Medicare across all racial/ethnic groups. Significant differences were seen across race/ethnicity in comorbidity scores, living on Oahu, and by sex.

**Table 1:** Descriptive Characteristics of People Aged 65 years or Older With a Diabetes-Related Potentially Preventable Hospitalization (DRPH) by Race/Ethnicity, Hawai‘i, December 2006–December 2010^a^

Characteristic	Chinese	Filipino	Native Hawaiian	Japanese	White	*P* Value^b^
**No. of patients**	101 (6.7)	307 (20.3)	270 (17.8)	545 (36.0)	292 (19.3)	NA
**No. of hospitalizations**	120 (6.6)	368 (20.3)	331 (18.2)	632 (34.8)	364 (20.1)	NA
**Female**	49 (48.5)	175 (57.0)	145 (53.7)	276 (50.6)	124 (42.5)	.008
**Payer**						
Medicaid	—c	—c	—c	0	—c	.11
Medicare	98 (97.0)	282 (91.9)	250 (92.6)	516 (94.7)	270 (92.5)	.23
Private	—c	20 (6.5)	17 (6.3)	24 (4.4)	20 (6.9)	.22
Other	0	—c	—c	—c	—c	.81
**Live on Oahu**	95 (94.1)	234 (76.2)	152 (56.3)	425 (78.0)	173 (59.3)	<.001
**Types of DRPH**						
Uncontrolled diabetes (DM)	—c	15 (4.1)	—c	22 (3.5)	22 (6.0)	.032
Short-term DM complications	17 (14.2)	40 (10.9)	11 (3.3)	59 (9.3)	35 (9.6)	.0009
Long-term DM complication	69 (57.5)	225 (61.1)	206 (62.2)	418 (66.1)	194 (53.3)	.003
Lower-extremity DM-related amputations	32 (26.1)	88 (23.9)	108 (32.5)	133 (21.0)	113 (31.0)	<.001
**Charlson comorbidity index (** 21 **), mean (SD)**	4.83 (3.08)	4.97 (3.15)	6.38 (3.49)	4.40 (3.11)	5.48 (3.13)	<.001
**Continuous age, mean (SD)**	78.9 (7.2)	76.8 (7.0)	73.3 (6.1)	79.7 (7.2)	75.4 (6.9)	<.001
**No. of hospitalizations per patient, mean (SD)**	1.2 (0.5)	1.2 (0.5)	1.2 (0.6)	1.2 (0.5)	1.2 (0.6)	.25

Abbreviation: NA, not applicable; DM, diabetes mellitus.

a Data are presented as no. (%) unless otherwise indicated. Source: Hawai‘i Health Information Corporation inpatient data (17).

b *P* values based on χ^2^ tests (or Fisher’s exact tests) for categorical variables and one-way analysis of variance for continuous variables except for number of hospitalizations per patient, for which Kruskal–Wallis test was used.

c This number is <10 and cannot be reported.

### Population totals as denominator

In unadjusted models, with population totals as the rate denominator, disparities in DRPH were seen for women and men in all AA/PI racial/ethnic groups compared with whites, with RRs ranging from 1.32 in Chinese men to 3.98 in Filipino women (Table 2). These findings remained when other factors were controlled. Even in the adjusted model (model A), a significantly higher population-level burden of DRPH was seen for women and men in all nonwhite racial/ethnic groups compared with whites. Adjusted RRs ranged from 1.94 (95% CI, 1.30–2.91) in Chinese women to 4.18 (95% CI, 3.31–5.27) in Filipino women.

**Table 2:** Unadjusted and Adjusted Rate Ratios of Diabetes-Related Potentially Preventable Hospitalizations (DRPH) for People Aged 65 Years or Older by Race/Ethnicity and Sex, Using Population Totals for the Denominator,^a^ Hawai‘i, December 2006–December 2010

Sex and Race/Ethnicity	Population Totals	No. of DRPH^b^	No. of Unique Individuals With DRPH^b^	Unadjusted Annual Rate per 10,000 Population	Unadjusted Rate Ratio per Patient by Population Totals	Adjusted Model by Population Totals	
						Adjusted Rate Ratio (95% CI)	*P* Value^c^
**Female**							
Chinese	7,726	59	49	15.53	1.69	1.94 (1.30–2.91)	.002
Filipino	11,700	209	175	36.63	3.98	4.18 (3.31–5.27)	<.001
Native Hawaiian	10,560	174	145	33.63	3.65	3.25 (1.90–5.27)	<.001
Japanese	40,186	310	276	16.82	1.83	2.96 (1.81–4.82)	<.001
White	33,005	151	124	9.20	Reference	Reference	Reference
**Male**							
Chinese	6,893	61	52	18.47	1.32	2.13 (1.11–4.08)	.024
Filipino	8,381	159	132	38.57	2.75	3.49 (2.64–4.62)	<.001
Native Hawaiian	7,063	157	125	43.34	3.09	2.57 (1.79–3.71)	<.001
Japanese	25,393	322	269	25.94	1.85	2.55 (1.40–4.63)	.003
White	29,366	213	168	14.01	Reference	Reference	Reference

Abbreviation: CI, confidence interval.

a For multivariate comparisons. Based on multivariable model adjusting for age (fixed at 77 y), race/ethnicity, sex, median Charlson Comorbidity Index (21) (fixed at 4), percentage of public insurer (fixed at 73%), and location of residence (fixed at Oahu); for the multivariable model, age, sex, race/ethnicity, and location of residence were significant at *P* value < .05.

b Data source: Hawai‘i Health Information Corporation (17).

c *P* values were obtained from multivariable negative binomial regression models.

### Diabetes prevalence as denominator

When the rates of DRPH by patient only among those with diabetes were considered, for some AA/PI groups, particularly women, the higher population-level burden of DRPH was explained by the higher diabetes burden in AA/PI groups compared with whites. When the number of people with diabetes was used as the denominator, even in unadjusted models, no disparity in DRPH was seen for Chinese women (RR = 0.90) and men (RR = 0.91) and Japanese women (RR = 0.95) relative to whites. Whites appear to have a disparity relative to these AA/PI groups (Table 3).

**Table 3:** Unadjusted and Adjusted Rate Ratios of Diabetes-Related Potentially Preventable Hospitalizations (DRPH) for People Aged 65 Years or Older by Race/Ethnicity and Sex, Using the Number With Diabetes for the Denominator,^a^ Hawai‘i, December 2006–December 2010

Sex and Race/Ethnicity	No. (%) With Diabetes^b^	No. of Unique Individuals With DRPH^c^	Unadjusted Annual DRPH Rate per 10,000 Patients With Diabetes	Unadjusted Rate Ratio by No. With Diabetes	Adjusted Model by No. with Diabetes	
					Adjusted Rate Ratio (95% CI)	*P* Value^d^
**Female**						
Chinese	1,424 (18.4)	49	84.27	0.90	0.91 (0.61–1.36)	.64
Filipino	2,939 (25.1)	175	145.82	1.56	1.61 (1.28–2.03)	<.001
Native Hawaiian	3,226 (30.6)	145	110.08	1.18	1.13 (0.67–1.89)	.66
Japanese	7,571 (18.8)	276	89.28	0.95	1.38 (0.85–2.25)	.20
White	3,242 (9.8)	124	93.67	Reference	Reference	Reference
**Male**						
Chinese	1,420 (20.6)	52	89.68	0.91	1.21 (0.64–2.30)	.57
Filipino	1,880 (22.4)	132	171.95	1.75	2.26 (1.71–2.99)	<.001
Native Hawaiian	1,707 (24.2)	125	179.33	1.82	1.59 (1.11–2.28)	.012
Japanese	4,383 (17.3)	269	150.30	1.53	1.86 (1.04–3.33)	.037
White	4,176 (14.2)	168	98.52	Reference	Reference	Reference

Abbreviation: CI, confidence interval.

a For multivariate comparisons. Based on multivariable model adjusting for age (fixed at 77 y), race/ethnicity, sex, median Charlson Comorbidity Index (21) (fixed at 4), percentage of public insurer (fixed at 73%), and location of residence (fixed at Oahu); age, sex, race/ethnicity, and location of residence were significant at *P* < .05.

b From Hawai‘i Department of Health Behavioral Risk Factor Surveillance System analysis.

c From Hawai‘i Health Information Corporation analysis.

d *P* values were obtained from multivariable negative binomial regression models.

However, this was not true for all AA/PI groups. Even when the higher prevalence of diabetes was considered, disparities in DRPH remained for Filipinos, Native Hawaiians, and Japanese in unadjusted models. In fully adjusted models, disparities remained for Filipino women (aRR = 1.61; 95% CI, 1.28–2.03) and Filipino men (aRR = 2.26; 95% CI, 1.71–2.99), Native Hawaiian men (aRR = 1.59; 95% CI, 1.11–2.28), and Japanese men (aRR = 1.86; 95% CI, 1.04–3.33) compared with whites.

## Discussion

Significant disparities in DRPH were seen among many AA/PI subpopulations compared with whites, in particular for men. Even in fully adjusted models among elderly adults, accounting for insurer, comorbidity, age, location of residence, and population-level diabetes, Native Hawaiian, Filipino, and Japanese men all had higher rates of DRPH than whites. Higher rates of DRPH were also seen among Filipino women compared with whites in final models.

This study adds new evidence about diabetes-related hospitalizations, a particularly expensive, burdensome, and often preventable consequence of diabetes. As expected, among elderly adults, we found disparities for Native Hawaiians and Filipinos compared with whites. Native Hawaiians and Filipinos are known to have lower access to primary care and to have higher rates of diabetes-related illness and death (2,12). We also found disparities among elderly Japanese men. This was unexpected, as Japanese in Hawai‘i tend to have favorable health profiles (26) and good access to, and use of, chronic care management. It would be interesting to consider whether these disparities are also seen among those aged less than 65 years to see if this is an age or a cohort effect. If diabetes management is getting worse among the Japanese as they age, this is a critical transition area in which to improve care. Also, as the largest total number of DRPH was among Japanese individuals, this is an important issue.

Our results confirm that disparities in DRPH exist in AA/PI subgroups, even among the elderly who are primarily insured under the same provider (Medicare). This has also been found among African Americans compared with whites (9). National and state-level programs targeted for groups with particular burdens of DRPH are needed.

This study adds to the growing evidence base showing the importance of disaggregating AA/PI subgroups to better understand specific health risks generally and for diabetes specifically (2,12,13). Previous research using combined AA/PI samples did not find significant disparities in DRPH compared with whites (10,11), but when AA/PI groups were disaggregated, we found significant differences for some elderly AA/PI groups compared with whites. Disparities also varied in magnitude between AA/PI groups, a finding that would similarly be hidden in aggregated AA/PI analyses.

We also provide further evidence that using population totals versus disease prevalence totals indicate different portraits of rates and of disparities in preventable hospitalizations (22). The large variation in the magnitude of racial/ethnic associations across the 2 multivariable models suggests that most disparities in DRPH are due to disparities in prevalence. However, this is not the case for all racial/ethnic groups. For example, when population totals were used as denominators, a higher prevalence of DRPH was seen in Japanese, Native Hawaiian, and Filipino women and men relative to whites even in adjusted models. However, once the higher diabetes prevalence in Japanese, Native, Hawaiian, and Filipino populations compared with whites was considered, the relative burden of DRPH disappeared for Japanese and Native Hawaiian women but not for Filipino women or for men of any of the 3 AA/PI racial groups. In these groups it was reduced but not eliminated.

Thus, the reasons for the higher DRPH in elderly adults among Filipinos, Japanese, and Native Hawaiians appear distinct by age and race/ethnicity and suggest differential interventions. To resolve the burden of DRPH among Japanese and Native Hawaiian women, reducing diabetes generally appears to be most important. Filipino women with diabetes, as well as Japanese, Native Hawaiian, and Filipino men, appear to have a further issue with access to care, some health care utilization factor, or more severe illnesses that lead to a double diabetes burden among these populations — higher rates of diabetes and a higher likelihood of being hospitalized with a preventable complication of diabetes.

By using the master patient ID information available in the HHIC data set, we were able to consider unique individuals. This allowed us to ensure that multiple visits by unique patients within racial/ethnic groups were not responsible for our findings, an acknowledged weakness in many previous studies on this topic and a particular issue in diabetes, where readmissions are known to vary by race/ethnicity (18).

### Limitations

Individuals had to have a diabetes diagnosis to meet the AHRQ definition for DRPH. Because 40% of diabetes in the United States is undiagnosed (27), some visits that should be classified as a DRPH may not be. Also, the self-reported diabetes prevalence obtained from the BRFSS likely underestimates true prevalence, both because not all people with diabetes are aware of their condition (which may vary by race/ethnicity) and because the BRFSS sample excludes people without a telephone, which may include people particularly likely to be hospitalized for diabetes (28). If whites and all AA/PI groups are similarly underestimated, the underestimation would affect point estimates, but not relative rates.

Although we have a comprehensive, state-level data set, we include only 1 state, and it may not be representative of other areas. However, because access to culturally appropriate care may be worse for AA/PI groups in many other settings, our study may actually underestimates AA/PI disparities.

Our analyses are based on administrative data, which have some general limitations (29) and lack some variables that would have been useful, such as demographics (eg, education), modifiable risk factors (eg, obesity), differences in access (eg, having a primary care provider), pathways to care (eg, emergency department vs outpatient setting), or quality of care. We also lack information about the time since a diabetes diagnosis. These are all areas for future research. Also, although the HHIC race/ethnicity data have been verified to be collected consistently across hospitals, this is not guaranteed in all cases. However, in this regard we share these limitations with many studies on this topic because the AHRQ metrics are designed to use administrative data.

### Conclusions

We identified 1,815 hospitalizations by 1,515 elderly patients in a 4-year period in Hawai‘i that were potentially avoidable with better primary care for diabetes. The burdens of these hospitalizations are distributed unevenly across AA/PI populations. The first step in reducing disparities in preventable hospitalizations is documenting such disparities. Although disparities between many other racial/ethnic groups in preventable hospitalizations have been noted (9), AA/PI subgroups have not been well represented in this research. As preventable hospitalizations have received considerable policy attention (8,30) and hidden AA/PI subgroup disparities are receiving increasing focus (2,12–14), this is a timely and important issue.

## References

[1] National Diabetes Fact Sheet. 2011. Fast facts on diabetes. Atlanta (GA): Centers for Disease Control and Prevention. http://www.cdc.gov/diabetes/pubs/pdf/ndfs_2011.pdf. Accessed December 5, 2012.

[2] King GL , McNeely MJ , Thorpe LE , Mau ML , Ko J , Liu LL , Understanding and addressing unique needs of diabetes in Asian Americans, Native Hawaiians, and Pacific Islanders. Diabetes Care 2012;35(5):1181–8. 10.2337/dc12-0210 22517939 PMC3329823

[3] State of Hawaii, Behavioral Risk Factor Surveillance System, from 2005 to 2007; by demographic characteristic and ethnic group. Honolulu (HI): Hawaii State Department of Health. http://hawaii.gov/health/statistics/brfss/ethnicity/0567/ethnicity567.html. Accessed December 6, 2012.

[4] McBean AM , Li S , Gilbertson DT , Collins AJ . Differences in diabetes prevalence, incidence, and mortality among the elderly of four racial/ethnic groups: whites, blacks, Hispanics, and Asians. Diabetes Care 2004;27(10):2317–24. 10.2337/diacare.27.10.2317 15451894

[5] Kanaya AM , Adler N , Moffet HH , Liu J , Schillinger D , Adams A , Heterogeneity of diabetes outcomes among Asians and Pacific Islanders in the US: the Diabetes Study of Northern California (DISTANCE). Diabetes Care 2011;34(4):930–7. 10.2337/dc10-1964 21350114 PMC3064053

[6] Karter AJ , Ferrara A , Liu JY , Moffet HH , Ackerson LM , Selby JV , Ethnic disparities in diabetic complications in an insured population. JAMA 2002;287(19):2519–27. Erratum in JAMA 2002;288(1):46. 10.1001/jama.287.19.2519 12020332

[7] Prevention Quality Indicators overview. Rockville (MD): Agency for Healthcare Research and Quality; 2011. http://www.qualityindicators.ahrq.gov/modules/pqi_overview.aspx. Accessed October 11, 2012.

[8] McCarthy D , How SKD , Schoen C , Cantor JC , Belloff D . Aiming higher: results from a state scorecard on health system performance, 2009. The Commonwealth Fund Commission on a High Performance Health System; 2009. http://www.commonwealthfund.org/~/media/Files/Publications/Fund%20Report/2009/Oct/1326_McCarthy_aiming_higher_state_scorecard_2009_full_report_FINAL_v2.pdf. Accessed October 21, 2011.

[9] Laditka JN , Laditka SB . Race, ethnicity and hospitalization for six chronic ambulatory care sensitive conditions in the USA. Ethn Health 2006;11(3):247–63. 10.1080/13557850600565640 16774877

[10] Russo C , Andrews R , Coffey R . Racial and ethnic disparities in potentially preventable hospitalizations, 2003. HCUP Statistical Brief #10; 2006. http://www.hcup-us.ahrq.gov/reports/statbriefs/sb10.pdf. Accessed March 31, 2011. 21938842

[11] Moy E , Barrett M , Ho K . Potentially preventable hospitalizations — United States, 2004–2007. MMWR Surveill Summ 2011;60 Suppl:80–3. 21430628

[12] Mau MK , Sinclair K , Saito EP , Baumhofer K , Kaholokula JK . Cardiometabolic health disparities in Native Hawaiians and other Pacific Islanders. Epidemiol Rev 2009;31:113–29. 10.1093/ajerev/mxp004 19531765 PMC2893232

[13] Ghosh C . *Healthy People 2010* and Asian Americans/Pacific Islanders: defining a baseline of information. Am J Public Health 2003;93(12):2093–8. 10.2105/AJPH.93.12.2093 14652340 PMC1448158

[14] Moy E , Mau M , Raetzman S , Barrett M , Miyamura J , Chaves K , Ethnic differences in potentially preventable hospitalizations among Asian Americans, Native Hawaiians, and other Pacific Islanders: implications for reducing health care disparities. Ethn Dis 2013;23(1):6–11. 23495615

[15] US Census Bureau. The Native Hawaiian and Other Pacific Islander population: 2010. Issued May 2012. http://www.census.gov/prod/cen2010/briefs/c2010br-12.pdf. Accessed July 25, 2012.

[16] US Census Bureau. Annual estimates of the resident population by sex, race, and Hispanic origin for the United States: April 1, 2000 to July 1, 2009; 2011. http://www.census.gov/popest/national/asrh/NC-EST2009-srh.html. Accessed October 21, 2011.

[17] Hawaii Health Information Corporation, inpatient data. http://hhic.org/inpatient-data.asp. Accessed March 31, 2011.

[18] Jiang HJ , Andrews R , Stryer D , Friedman B . Racial/ethnic disparities in potentially preventable readmissions: the case of diabetes. Am J Public Health 2005;95(5):1561–7. 10.2105/AJPH.2004.044222 16118367 PMC1449398

[19] Prevention Quality Indicators resources. Rockville (MD): Agency for Healthcare Research and Quality; 2011. http://www.qualityindicators.ahrq.gov/Modules/pqi_resources.aspx. Accessed October 11, 2012.

[20] American Diabetes Association. Data from the 2011 National Diabetes Fact Sheet (released Jan. 26, 2011); 2011. http://www.diabetes.org/diabetes-basics/diabetes-statistics/. Accessed June 25, 2012.

[21] Charlson ME , Pompei P , Ales KL , MacKenzie CR . A new method of classifying prognostic comorbidity in longitudinal studies: development and validation. J Chronic Dis 1987;40(5):373–83. 10.1016/0021-9681(87)90171-8 3558716

[22] Lui CK , Wallace SP . A common denominator: calculating hospitalization rates for ambulatory care-sensitive conditions in California. Prev Chronic Dis 2011;8(5):A102. 21843405 PMC3181231

[23] Wang J , Imai K , Engelgau MM , Geiss LS , Wen C , Zhang P . Secular trends in diabetes-related preventable hospitalizations in the United States, 1998–2006. Diabetes Care 2009;32(7):1213–7. 10.2337/dc08-2211 19366966 PMC2699731

[24] Pobutsky A , Balabis J , Ngueyen D-H , Tottori C . Hawai‘i diabetes report 2010. Honolulu (HI): Hawai‘i State Department of Health, Chronic Disease Management and Control Branch, Diabetes Prevention and Control Program; 2011. http://www.Hawaiihealthmatters.org/javascript/htmleditor/uploads/DOH_DiabetesReport2010_Lo.pdf. Accessed June 25, 2012.

[25] Long JS , Freese J . regression models for categorical dependent variables using Stata. Second edition. College Station (TX): Stata Press; 2006.

[26] Park CB , Braun KL , Horiuchi BY , Tottori C , Onaka AT . Longevity disparities in multiethnic Hawaii: an analysis of 2000 life tables. Public Health Rep 2009;124(4):579–84. 19618795 10.1177/003335490912400415 PMC2693172

[27] Cowie CC , Rust KF , Ford ES , Eberhardt MS , Byrd-Holt DD , Li C , Full accounting of diabetes and pre-diabetes in the U.S. population in 1988–1994 and 2005–2006. Diabetes Care 2009;32(2):287–94. 10.2337/dc08-1296 19017771 PMC2628695

[28] Nelson DE , Holtzman D , Bolen J , Stanwyck CA , Mack KA . Reliability and validity of measures from the Behavioral Risk Factor Surveillance System (BRFSS). Soz Praventivmed 2001;46 Suppl 1:S3–42. 11851091

[29] Billings J . Using administrative data to monitor access, identify disparities, and assess performance of the safety net: tools for monitoring the health care safety net; 2003. http://archive.ahrq.gov/data/safetynet/billings.htm. Accessed June 12, 2013.

[30] National healthcare disparities report 2010. Chapter 7: efficiency. Bethesda (MD): Agency for Healthcare Research and Quality; 2010. http://www.ahrq.gov/research/findings/nhqrdr/nhdr10/Chap7.html. Accessed June 12, 2013.

