# Erratum, Vol. 10, July 25 Release

**DOI:** 10.5888/pcd10.120340e

**Published:** 2013-08-29

In the article “Comparison of Potentially Preventable Hospitalizations Related to Diabetes Among Native Hawaiian, Chinese, Filipino, and Japanese Elderly Compared with Whites, Hawai‘i, December 2006–December 2010,” an incorrect number was given in the results section of the Abstract. This sentence should have read “Unadjusted RRs for DRPH by patient were greater than 1 in all AA/PI study groups compared with whites, but were highest among Native Hawaiians and Filipinos.” The correction was made to our website on August 8, 2013, and appears online at http://www.cdc.gov/pcd/issues/2013/12_0340.htm. We regret any confusion or inconvenience this error may have caused.

